# Predictive and Prognostic Role of Systemic Immune-Inflammation Index (SII) in Metastatic Colorectal Cancer Patients Treated with Trifluridine/Tipiracil

**DOI:** 10.3390/biomedicines12092076

**Published:** 2024-09-11

**Authors:** Mateusz Malik, Barbara Radecka, Marek Gełej, Aleksandra Jackowska, Emilia Filipczyk-Cisarż, Michalina Żurowska, Katarzyna Hetman, Małgorzata Foszczyńska-Kłoda, Beata Kania-Zembaczyńska, Danuta Mańka, Marlena Orlikowska, Lubomir Bodnar

**Affiliations:** 1 Clinical Oncology Department, Lower Silesian Oncology, Pulmonology and Hematology Center, 53-413 Wroclaw, Poland; cisarz.emilia@dco.com.pl; 2 Department of Oncology, Institute of Medical Sciences, University of Opole, 45-040 Opole, Poland; brad@onkologia.opole.pl (B.R.);; 3 Department of Clinical Oncology, Tadeusz Koszarowski Cancer Center in Opole, 45-061 Opole, Poland; 4 Oncology and Immunooncology Clinic, Warmia and Mazury Oncology Centre, MSWiA Hospital, 10-228 Olsztyn, Poland; 5 Department of Clinical Oncology and Radiotherapy, St. John Paul II Mazovia Regional Hospital in Siedlce, 08-110 Siedlce, Polandlubomirbodnar.lb@gmail.com (L.B.); 6 Department of Clinical Oncology, West Pomeranian Oncology Center in Szczecin, 71-730 Szczecin, Poland; khetman@onkologia.szczecin.pl (K.H.); mfoszczynska@onkologia.szczecin.pl (M.F.-K.); 7 Department of Oncology and Oncohematology, Beskid Oncology Centre in Bielsko-Biala, 43-300 Bielsko Biala, Poland; 8 Oncology Department, Kociewie Health Centre, 83-200 Starogard Gdanski, Poland; 9 Faculty of Medical and Health Sciences, Siedlce University of Natural Sciences and Humanities, 08-110 Siedlce, Poland

**Keywords:** cancer, systemic inflammation markers, chemotherapy, mCRC, prognosis

## Abstract

In advanced-stage colorectal cancer (CRC), a strategy based on a sequence of systemic therapies brings survival benefits in most patients. Trifluridine and tipiracil hydrochloride (TT) is a chemotherapy drug effective in patients in the third- or later line setting. No highly specific biomarkers have been established for TT therapy so far. However, a systemic immune-inflammation index (SII), which is based on platelet, neutrophil and lymphocyte counts is applied to predict prognosis. In this retrospective, multicenter study, clinical data on 179 metastatic CRC patients treated with TT were collected. To evaluate factors predicting TT therapy response and overall survival, univariate logistic regression analysis was conducted. Subsequently, factors with *p* < 0.05 in univariate analysis were included in multivariate analysis. In the multivariate analysis of progression-free survival (PFS), three favorable parameters were significant: good to moderate histological differentiation (*p* = 0.0038), carcinoembryonic antigen (CEA) < 5 ng/L (*p* = 0.0316) and SII ≤ 550 (*p* = 0.007). Favorable prognostic factors revealed in the multivariate analysis of overall survival (OS) were: <3 prior lines of treatment (*p* = 0.02), good to moderate histological differentiation (*p* = 0.0003), CEA < 5 ng/L (*p* = 0.0227) and SII ≤ 550 (*p* = 0.0001). Our study indicated that pre-treatment SII may be clinically useful for selecting likely responder patients and assessing the prognosis for mCRC patients treated with TT.

## 1. Introduction

Colorectal cancer (CRC) is the third most frequent malignant disease around the world, with 1.9 million new cases annually. It is the third and the second most common type of malignancy in men in women, respectively [1]. CRC is responsible for approximately 8.9% of cancer-related deaths, a figure that is steadily increasing. Overall, the 5-year cumulative survival of patients with CRC is 64%, but decreases to 15% in those with the advanced disease [2]. Poland was among the top ten countries with the highest overall mortality rate from colorectal cancer in 2020 [3]. 

The combination of trifluridine and tipiracil hydrochloride (TT) comprises a thymidine-based nucleoside analog, trifluridine, and a thymidine phosphorylase inhibitor, tipiracil. TT, as cytostatic antimetabolite drug, after being metabolized in cancer cells to the triphosphate form, is incorporated directly into DNA, which prevents cell proliferation. Additionally, another metabolite—monophosphate—reversibly inhibits thymidine synthase, which leads to an imbalance between deoxythymidine triphosphate and thymidine monophosphate, resulting in the interruption of the DNA structure. To prevent the drug resistance of trifluridine that is affected by thymidine phosphorylase, the combination contains its inhibitor—tipiracil. The efficacy and safety of TT in patients with metastatic CRC (mCRC) refractory to standard therapies were evaluated in the phase III RECOURSE trial [4]. TT, along with regorafenib and fruquintinib, is one of the few drugs whose effectiveness in the group of the patients resistant to the standard treatment—fluoropyrimidine, oxaliplatin, irinotecan, vascular endothelial growth factor (VEGF) inhibitor and/or epidermal growth factor receptor (EGFR) inhibitor in case of *RAS* wild-type status—has been confirmed in randomized trials [5,6]. The combination of TT with bevacizumab has significantly improved the effectiveness of chemotherapy (CT), but a significant number of patients are still treated with TT monotherapy due to contraindications to anti-VEGF therapy or financial/organizational constraints [7]. A number of other conventional chemotherapeutic agents, such as capecitabine, mitomycin C and gemcitabine, are an option as salvage therapy in mCRC, but their beneficial effects in the third- or later line setting are limited. In some patients, oxaliplatin/irinotecan or anti-EGFR rechallenge therapy might be an option [8]. For mCRC patients with high microsatellite instability (MSI-H) or deficient DNA mismatch repair (dMMR), the anti-programmed cell death protein 1 (anti-PD-1) immune checkpoint inhibitors (CPIs) in monotherapy or in combination with anti-cytotoxic T-lymphocyte antigen 4 (anti-CTLA-4) antibodies are also approved in the second- or later line setting [9]. 

The prognosis for ‘heavily pretreated’ patients is poor. For patients who received a first line of anticancer therapy, the probabilities to receive further lines are as follows: 74.3% for the second, 47.0% for the third and 21.6% for the fourth. Nevertheless, later lines could be beneficial even though earlier ones were not [10]. Most patients reveal disease symptoms or suffer from previous systemic treatment toxicities. Those with optimal performance status, who have recovered from previous complications, can be offered late-line CT on top of best supportive care (BCS) in order to prolong survival with acceptable quality of life. 

It has been extensively documented that inflammatory cells and mediators are present in the microenvironment of most, if not all, cancers, irrespective of the trigger. As the number of peripheral inflammatory cells reflects the status of immune response in cancer patients, several inflammation- and immune-based prognostic scores have been developed to predict cancer prognosis, such as the Glasgow Prognostic Score (GPS), tumor-related leukocytosis (TRL), neutrophil-to-lymphocyte ratio (NLR), platelet-to-lymphocyte ratio (PLR) and lymphocyte-to-monocyte ratio (LMR, sometimes referred to as MLR) or systemic immune-inflammation index (SII) [11,12,13,14,15,16]. Furthermore, some of them (NLR, PLR and SII) have been identified as pre-diagnostic markers of CRC risk [17]. 

SII, a relatively novel clinical tool, consists of platelet, neutrophil and lymphocyte counts. It was first described for prognosis assessment in hepatocellular carcinoma [15]. A normalized cut-off value has not been established and varies for different cancer types, but to date a high negative prognostic value of SII has been observed in multiple cancers, including CRC, and also in some non-cancerous diseases [18,19,20,21]. Unlike the prognostic value of SII, its predictive value for response to systemic treatments—including CT, targeted therapies and CPI immunotherapy—in cancer patients has been the subject of a small yet increasing number of publications [22,23,24]. 

No specific biomarkers have been established for TT CT so far. The aim of this study was to investigate the predictive (affecting PFS) and prognostic (affecting OS) role of SII in the context of other prognostic factors in metastatic CRC patients treated with TT.

## 2. Materials and Methods

### 2.1. Patients

We retrospectively analyzed a group of 179 colorectal cancer patients. The inclusion criteria were as follows: (1) confirmed histopathological diagnosis of CRC; age > 18 years; (2) advanced stage of disease (de novo or after radical treatment in the past); (3) presence of measurable lesions determined by Response Evaluation Criteria in Solid Tumors, version 1.1 [25]; (4) at least one prior treatment regimen for advanced disease before TT onset; (5) disease progression during or after fluoropyrimidine, oxaliplatin and irinotecan CT combined with anti-VEGF therapy (if available) and/or anti-EGFR therapy (in case of RAS wild-type status); (6) performance status ≤2 according to the ECOG scale; (7) at least 1 cycle of TT received.

The exclusion criteria were as follows: (1) history of a second malignant tumor, except those previously subjected to radical treatment; (2) lack of access to complete medical records or lack of consent to provide them.

All source data were collected from 7 cancer sites in Poland from the medical records of all the patients meeting the above criteria between 2017 and 2021. Data provided by each site were blinded before the analysis. The study was approved by the decision #18/2019/VII of the Warmian-Masurian Medical Chamber Ethics Committee in Olsztyn (Poland).

### 2.2. Parameters and Statistical Analysis

The value of SII was determined from the equation: SII = P × N/L, where P, N and L are the baseline peripheral blood platelet, neutrophil and lymphocyte counts per liter, respectively. The SII cut-off values, low: ≤550 (×10^9^ cells/L) and high: >550 (×10^9^ cells/L), were selected based on the meta-analysis by Dong et al. as the most representative for CRC patients [26]. Progression-free survival (PFS) was determined from the date of treatment initiation to the date of disease progression defined by the RECIST 1.1 criteria (if available) or the date of the last follow-up. Overall survival (OS) was determined from the date of treatment initiation to the date of death or the date of final follow-up. The cut-off date for our analysis was set on 5 July 2021. Variables whose differences had a significant impact on PFS were considered predictive factors. Variables whose differences had a significant impact on OS were considered prognostic factors. Univariate analyses of variables affecting PFS or OS were performed by log-rank test; this identified a preliminary list of significant factors. The variables found significant or showing a trend towards significance (*p* < 0.1) in the univariate analysis were included in the multivariate analysis. The multivariate analyses of PFS and OS were performed by the Cox proportional hazards regression model using the forward stepwise method. Medians and life tables were computed using the product limit estimate of the Kaplan and Meier method. Finally, the statistical significance was assessed by the log-rank test; *p*-values less than 0.05 were considered to indicate statistical significance. The statistical package MedCalc (ver. 19.7.2; MedCalc Software Ltd., Ostend, Belgium) was used for the analysis.

## 3. Results

### 3.1. Participants and Descriptive Data

The whole cohort of 179 patients was eligible for the final analysis. The cohort was heterogeneous: median age was 65 years (30 to 83), 62% of subjects were male, and all subjects were Caucasian. Predominantly, Eastern Cooperative Oncology Group (ECOG) performance status (PS) was 1 (80%), primary tumor location was left-sided (81%), and histological subtype was adenocarcinoma (96%) with moderate histological differentiation (65%). The median of previous lines of treatment for advanced disease was 2 (1 to 6). Median SII was 615 (136 to 6048) at treatment onset. Detailed patient characteristics are presented in Table 1. 

TT was administered until the clinical and/or radiological progression of the disease. The median number of TT cycles was 3 (1 to 15). The median follow-up period was 16.6 months (95% CI, 14.7 to 37.2), and 72.1% of patients (129/179) had died by the end thereof. The clinical benefit ratio was 43% for best response, 0% for complete response, 3.9% for partial response, 39.1% for stable disease and 55.3% for progressive disease, according to Response Evaluation Criteria In Solid Tumors (RECIST), version 1.1; with 1.7% of not assessed. 

### 3.2. Outcome Data

Median PFS was 3.59 months (95% CI, 3.0 to 18.6) in the study cohort.

Median OS was 9.97 months (95% CI, 8.6 to 11.6).

### 3.3. Main Results

Univariate analysis indicated some potential favorable predictive parameters for TT therapy, namely: good to moderate histological differentiation (*p* = 0.0163), absence of liver metastases (*p* = 0.0103), NLR ≤ 3 (*p* = 0.0283), PLR ≤ 150 (*p* = 0.0019), SII ≤ 550 (*p* = 0.0070) and carcinoembryonic antigen (CEA) <5 ng/L (*p* = 0.0008). 

**Figure 1:**
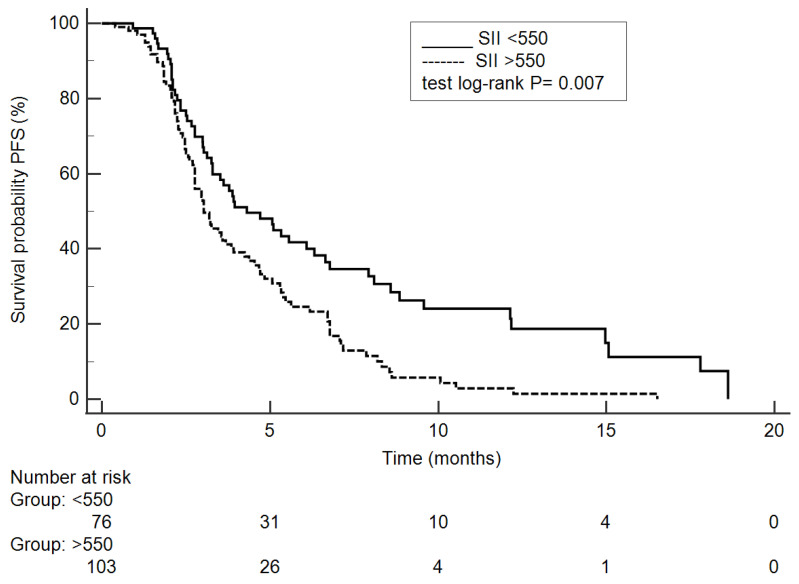
Kaplan–Meier curves showing progression-free survival under trifluridine/tipiracil adjusted to SII ≤ 550 or >550.

In the multivariate analysis of PFS, three parameters were found to be significant: good to moderate histological differentiation (*p* = 0.0038), CEA < 5 ng/L (*p* = 0.0316) and SII ≤ 550 (*p* = 0.0071). The results of PFS analysis are presented in Table 2 and Table 3, and Figure 1.

Univariate analysis identified several prognostic factors for OS improvement, namely: number of previous lines of treatment ≥3 (*p* = 0.024), good to moderate histological differentiation (*p* = 0.0055), NLR ≤ 3 (*p* = 0.0217), PLR ≤ 150 (*p* = 0.011), SII ≤ 550 (*p* = 0.0004) and CEA < 5 ng/L (*p* = 0.0003).

**Figure 2:**
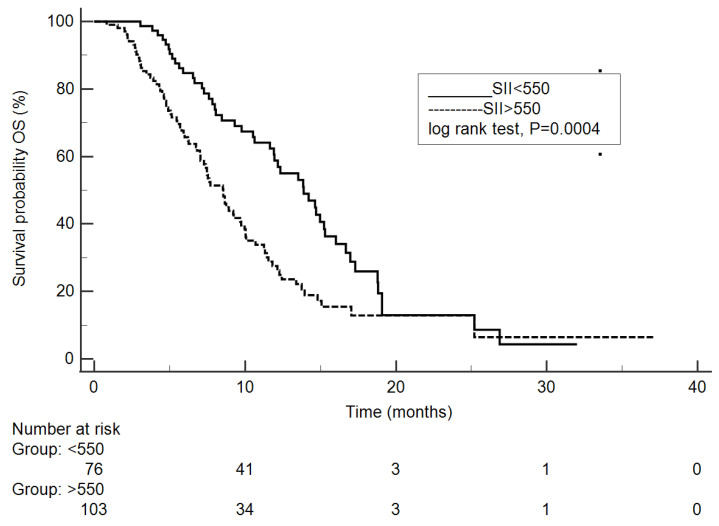
Kaplan–Meier curves showing overall survival under trifluridine/tipiracil adjusted to SII ≤ 550 or >550.

Multivariate analysis revealed four significant factors for survival improvement: number of prior lines of treatment <3 (*p* = 0.02), good to moderate histological differentiation (*p* = 0.0003), CEA < 5 ng/L (*p* = 0.0227) and SII ≤ 550 (*p* = 0.0001). The results of OS analysis are presented in Table 4 and Table 5, and Figure 2.

## 4. Discussion

The link between inflammation and different types of cancer has been confirmed. Immune cells play an essential role in production of cytokines that may promote or inhibit cancer proliferation, angiogenesis and metastasis, but also affect response to systemic therapies [11]. Consequently, the number and type of cells detectable in the blood count reflects the complex balance between immune cells and cytokines. Three coefficients of SII play a relevant role both in carcinogenesis and antitumor response. In addition to their known physiological role, neutrophils may be manipulated by tumors to promote disease progression through deregulation of growth factors [27], while platelets influence multistep development of tumors by transmitting proliferative signals, decreasing apoptosis, inducing angiogenesis and facilitating metastasis [28]. The role of different subtypes of lymphocytes in antitumor innate and adaptive immunity is complex. Indeed, natural killer (NK) cells mediate oncolytic activity against a variety of tumor targets, and cytotoxic T lymphocytes (CTLs) induce the tumor-associated antigen and IFN-γ production that affects cell cycle arrest, apoptosis, angiostasis and tumoricidal macrophage activity [29]. Generally, a shift from the immune response towards the inflammatory response, represented by high neutrophil and platelet levels as well as lymphopenia, is considered prognostically unfavorable in cancer patients. As a combination of PLR and NLR, SII could widely reflect both the inflammatory activity of the cancer and the immune response of the host. Apparently, the chronic systemic inflammatory response is associated with nutritional and functional deterioration and subsequent poor outcomes in cancer patients. Thus, systemic inflammation-based prognostic scores, like SII, can identify patients at risk [30]. 

A growing number of publications have described the prognostic role of SII in cancer, including CRC. Two independent meta-analyses published in 2020 explore the prognostic and predictive value of SII in CRC [26,31]. The first study, by Dong et al. (a meta-analysis of 12 studies with a total of 3919 CRC patients undergoing surgical resection and/or receiving systemic treatment in neo/adjuvant or palliative setting), indicates that high SII levels were significantly associated with lower PFS (HR = 1.74, 95% CI = 1.26–2.39, *p* = 0.001) and OS (HR = 1.61, 95% CI = 1.21–2.13, *p* = 0.001) in CRC. Moreover, elevated SII itself was found to be correlated with poor tumor differentiation, the presence of distant metastasis, an ECOG PS of 1–2 and a tumor size ≥5 cm, in patients with CRC, which are clinical factors implying a higher malignancy of the disease. SII cut-off values ranged from 340 to 1505. 

Regarding the predictive value of inflammatory markers in CRC, Eraslan et al. investigated the relationship between NLR, PLR, LMR, SII and the prognostic nutritional index (PNI), and pathological complete response (pCR) in patients with locally advanced rectal cancer (LARC) who received neoadjuvant chemoradiotherapy. Only SII was found to be an independent predictive factor of pCR (OR: 0.471, 95% CI; 0.224–0.991, *p* = 0.047). SII cut-off value was <748 [32]. 

One of the biggest studies analyzing pre-treatment inflammation and immune-based scores as predictors of treatment efficacy in mCRC to date was published by Passardi et al. In the overall population of 289 patients, PFS and OS were higher if SII < 730 (*p* = 0.015 and 0.002, respectively), NLR < 3 (*p* = 0.0001 and <0.0001, respectively) and PLR < 169 (*p* = 0.004 and 0.008, respectively). Also, in the prospective ITACa trial, it was concluded that pre-treatment NLR is a good predictive marker for mCRC patients who are candidates for the first-line CT with or without bevacizumab (BEV). Patients with NLR < 3 in the CT plus BEV arm had higher PFS than those treated with CT alone (HR = 0.69, *p* = 0.021). There was no significant difference if NLR was elevated [22]. 

Our findings remain consistent with the results of the aforementioned studies. Low SII seems to be associated with improved PFS and OS also in the heavily pretreated mCRC patient subgroup. However, to the best of our knowledge, no study evaluating predictive and prognostic role of SII in TT therapy in CRC has been published to date. Interestingly, three other clinical parameters revealed significant positive values, both predictive and prognostic, in our multivariate analysis: good to moderate histological differentiation, absence of liver metastases and CEA < 5 ng/L. Nevertheless, as with most CT agents, it is difficult to select highly specific predictors due to the non-specific mechanism of action of cytostatics. 

The recent phase IIIb PRECONNECT study by Taieb et al., which evaluated the safety and efficacy of TT monotherapy in a large cohort of 914 patients, also identified some predictive factors associated with longer PFS: time since diagnosis of the first metastasis ≥ 18 months, <3 metastatic sites, baseline ECOG PS = 0, absence of liver metastasis, no previous treatment with regorafenib, baseline NLR < 5, normal baseline alkaline phosphatase, normal baseline aspartate aminotransferase, baseline albumin ≥ 35 g/L and baseline white blood cell count < 10 × 109/L. The authors also confirmed the positive predictive value of neutropenia during TT treatment: their analysis indicated a significant association between PFS and neutropenia occurrence over time (hazard ratio [HR]: 0.36 [95% CI: 0.31, 0.42]). The PRECONNECT study did not assess OS, and thus the related prognostic factors [33]. 

We have found some other studies concerning the predictive value of inflammation- and immune-based scores for TT in monotherapy or TT in combination with BEV. A single-center retrospective study by Matsuda et al. on a group of 33 patients suggests the value of NLR < 5 as a predictive and prognostic factor in patients on TT monotherapy. Also, PLR < 173.2 and ECOG PS < 2 were found to be good discriminators in relation to PFS and OS. Kuramochi et al. explored the predictive and prognostic value of pre-treatment NLR, PLR and LMR in mCRC patients undergoing TT + BEV as third-line treatment. post hoc analysis of this prospective phase II study with a cohort of 32 patients revealed a significantly higher disease control rate (87.5 vs. 43.8%), mPFS (4.9 vs. 2.3 m) and mOS (21.0 vs. 6.1 m) in the high-LMR group compared to the low-LMR group, respectively. The authors considered LMR as a valuable predictive and prognostic biomarker [34,35]. 

Our study presents some limitations related to the sample size and retrospective nature of the analysis. Nevertheless, we have demonstrated SII to be a promising clinical predictive and prognostic biomarker. Although this needs further research, SII can be easily incorporated into clinical practice in the context of expanding treatment options in pretreated mCRC patients.

## 5. Conclusions

In conclusion, our study indicated that pre-treatment SII is a valid predictive and prognostic biomarker for mCRC patients treated with TT and may be clinically useful for selecting likely responder patients and assessing the prognosis.

## Figures and Tables

**Table 1:** Patient characteristics.

Characteristics	*n*	*%*
Enrolled	179	100
Sex		
Male	111	62
Female	68	38
Age, years median (range)	65 (30–83)	
ECOG performance status		
0	27	15
1	143	80
2	9	5
Primary site		
Cecum	16	9
Ascending colon	5	3
Hepatic flexure of the large intestine	5	3
Transverse colon	8	5
Splenic flexure of the large intestine	6	3
Colon descending	6	3
Sigmoid	40	22
Rectosigmoid junction	25	14
Rectum	68	38
Primary tumor location		
Right-sided	34	19
Left-sided	145	81
Primary tumor histological subtype		
Adenocarcinoma	173	96
Mucinous adenocarcinoma	5	3
Signet ring cell carcinoma	1	1
Histological differentiation:		
Well	27	15
Moderate	116	65
Poorly	14	8
Unknown	22	12
pT category		
pT1	5	3
pT2	17	10
pT3	92	51
pT4	49	27
Not operated on	16	9
pN category		
pN0	34	19
pN1	68	38
pN2	57	32
pN3	2	1
Not operated on	18	10
Site of metastasis		
Liver	135	75
Lung	73	41
Lymph node	36	20
Peritoneum	23	13
*KRAS* mutation status		
Wild-type	89	50
Mutant	85	47
Not available	5	3
*BRAF* mutation status		
Wild-type	153	85
Mutant	11	6
Not available	1	1
*NRAS* mutation status		
Wild-type	166	93
Mutant	6	3
Not available	7	4
Number of prior lines of treatment		
1	2	1
2	100	56
3	61	34
4	12	7
5	3	2
6	1	1
BMI median, range (kg/m^2^)	27 (16–43)	
<25	56	31
≥25	123	69
CEA median, (95% CI) (ng/L)	86.0 (53.7–116.1)	
<30	134	75
≥30	45	25
NLR median, (95% CI), (×10^9^/L)	2.6 (2.4–2.9)	
≤3	149	83
>3	30	17
PLR median, (95% CI), (×10^9^/L)	147 (131–165)	
≤150	90	50
>150	89	50
SII median, (95% CI), (×10^9^/L)	615 (558–753)	
≤550	76	42
>550	103	58

Abbreviation: ECOG: Eastern Cooperative Oncology Group, pT: size or direct extent of the primary tumor given by histopathologic examination of a surgical specimen according to the TNM staging system, pN: degree of spread to regional lymph nodes given by histopathologic examination of a surgical specimen, BMI: body mass index, CEA: carcinoembryonic antigen, NLR: neutrophil-to-lymphocyte ratio, PLR: platelet-to-lymphocyte ratio, SII: systemic immune-inflammation index, CI: confidence interval.

**Table 2:** Univariate analysis of progression-free survival (log-rank test).

Covariate	*n* (%)	Median (Months)	*p* Value
Age			0.6938
≤70 year	139 (78%)	3.6	
>70 year	40 (22%)	3.4	
Gender			0.1570
Male	111 (62%)	3.4	
Female	68 (38%)	3.9	
Performance status (ECOG)			0.5552
0–1	170 (95%)	3.5	
2	9 (5%)	3.7	
Liver metastases:			0.0103 *
No	44 (25%)	4.7	
Yes	135 (75%)	3.2	
Lung metastases:			0.1300
No	106 (59%)	4.8	
Yes	73 (41%)	3.2	
Primary tumor location:			0.5933
Left-sided	145 (81%)	3.5	
Right-sided	34 (19%)	3.5	
Number of prior lines of treatment:			0.9651
<3	102 (57%)	3.7	
≥3	77 (43%)	3.3	
Histological differentiation:			0.0163 *
Well/moderate	36 (20%)	3.9	
Poorly/unknown	143 (80%)	2.4	
*KRAS*			0.7737
Wild-type	89 (50%)	3.0	
Mutated	85 (47%)	3.6	
*NRAS*			0.1476
Wild-type	166 (93%)	3.6	
Mutated	6 (3%)	3.2	
*BRAF*			0.4907
Wild-type	153 (85%)	3.6	
Mutated	11 (6%)	2.9	
NLR			0.0283 *
≤3	149 (83%)	3.8	
>3	30 (17%)	2.6	
PLR			0.0019 *
≤150	90 (50%)	4.6	
>150	89 (50%)	3.0	
SII			0.0070 *
≤550	76 (42%)	4.4	
>550	103 (58%)	3.0	
CEA			0.0008 *
<5 ng/L	14 (8%)	5.5	
≥5 ng/L	164 (92%)	3.3	
BMI			0.8853
<25	56 (31%)	3.0	
≥25	123 (69%)	3.7	

Abbreviations: ECOG: Eastern Cooperative Oncology Group, CEA: carcinoembryonic antigen, NLR: neutrophil-to-lymphocyte ratio, PLR: platelet-to-lymphocyte ratio, SII: systemic immune-inflammation index, BMI: body mass index, * statistically significant (*p* < 0.05).

**Table 3:** Multivariate analysis of progression-free survival.

Parameter	*p* Value	HR	HR 95% Lower	HR 95% Upper
SII	0.0071 *	1.62	1.14	2.31
≤550				
>550				
CEA	0.0316 *	2.21	1.07	4.56
<5 ng/L				
≥5 ng/L				
Histological differentiation:	0.0038 *	1.84	1.22	2.78
Well/moderate				
Poorly/unknown				
NLR	NS	NS	NS	NS
≤3				
>3				
PLR	NS	NS	NS	NS
≤150				
>150				
Liver metastases:	NS	NS	NS	NS
No				
Yes				

Abbreviations: SII: systemic immune-inflammation index, CEA: carcinoembryonic antigen; NLR: neutrophil-to-lymphocyte ratio, PLR: platelet-to-lymphocyte ratio, HR: hazard ratio, * statistically significant (*p* < 0.05).

**Table 4:** Univariate analysis of overall survival (log-rank test).

Covariate	*n* (%)	Median (Months)	*p* Value
Age			0.9918
≤70 year	139 (78%)	10.0	
>70 year	40 (22%)	7.6	
Gender			0.6764
Male	111 (62%)	10.0	
Female	68 (38%)	8.6	
Performance status (ECOG)			0.8483
0–1	170 (95%)	9.9	
2	9 (5%)	7.7	
Liver metastases:			0.4444
No	44 (25%)	10.0	
Yes	135 (75%)	10.1	
Lung metastases:			0.3097
No	106 (59%)	12.1	
Yes	73 (41%)	8.7	
Primary tumor location:			0.1549
Left-sided	145 (81%)	10.5	
Right-sided	34 (19%)	7.5	
No. of prior lines of treatment:			0.0240 *
<3	102 (57%)	8.7	
≥3	77 (43%)	11.7	
Histological differentiation:			0.0055 *
Well/moderate	36 (20%)	10.7	
Poorly/unknown	143 (80%)	6.8	
*KRAS*			0.2439
Wild-type	89 (50%)	10.0	
Mutated	85 (47%)	9.5	
*NRAS*			0.8076
Wild-type	166 (93%)	10.0	
Mutated	6 (3%)	NR	
*BRAF*			0.1005
Wild-type	153 (85%)	10.7	
Mutated	11 (6%)	6.5	
NLR			0.0217 *
≤3	149 (83%)	10.5	
>3	30 (17%)	6.2	
PLR			0.0110 *
≤150	90 (50%)	11.9	
>150	89 (50%)	8.8	
SII			0.0004 *
≤550	76 (42%)	13.9	
>550	103 (58%)	8.4	
CEA			0.0003 *
<5 ng/L	14 (8%)	17.7	
≥5 ng/L	164 (92%)	9.4	
BMI			0.6875
<25	56 (31%)	8.1	
≥25	123 (69%)	10.6	

Abbreviations: ECOG: Eastern Cooperative Oncology Group, CEA: carcinoembryonic antigen; * statistically significant (*p* < 0.05); NLR: neutrophil-to-lymphocyte ratio, PLR: platelet-to-lymphocyte ratio, SII: systemic immune-inflammation index, BMI: body mass index.

**Table 5:** Multivariate analysis of overall survival.

Parameter	*p* Value	HR	HR 95% Lower	HR 95% Upper
No. of prior lines of treatment:	0.0204 *	1.54	1.07	2.21
≥3				
<3				
Histological differentiation:	0.0003 *	2.15	1.42	3.28
Well/moderate				
Poorly/unknown				
SII	0.0001 *	2.12	1.46	3.08
≤550				
>550				
CEA	0.0227 *	2.64	1.15	6.10
<5 ng/L				
≥5 ng/L				
NLR	NS	NS	NS	NS
≤3				
>3				
PLR	NS	NS	NS	NS
≤150				
>150				

Abbreviations: SII: systemic immune-inflammation index, CEA: carcinoembryonic antigen; NLR: neutrophil-to-lymphocyte ratio, PLR: platelet-to-lymphocyte ratio, HR: hazard ratio, * statistically significant (*p* < 0.05).

## Data Availability

The data that support the findings of this study are available from the corresponding author upon reasonable request.
